# Single-photon emission from single InGaAs/GaAs quantum dots grown by droplet epitaxy at high substrate temperature

**DOI:** 10.1186/1556-276X-7-493

**Published:** 2012-08-31

**Authors:** Mohamed Benyoucef, Verena Zuerbig, Johann Peter Reithmaier, Tim Kroh, Andreas W Schell, Thomas Aichele, Oliver Benson

**Affiliations:** 1 Institute of Nanostructure Technologies and Analytics (INA), CINSaT, University of Kassel, Heinrich-Plett-Strasse 40, Kassel, 34132, Germany; 2 Nano-Optik, Humboldt-Universität zu Berlin, Newtonstrasse 15, Berlin, 12489, Germany

**Keywords:** III-V semiconductors, Quantum dots, Droplet epitaxy, Single-photon emission, Radiative lifetime, 87.57.uh, 78.67.Hc, 78.55.-m, 42.50.-p

## Abstract

The authors report single-photon emission from InGaAs quantum dots grown by droplet epitaxy on (100) GaAs substrates using a solid-source molecular beam epitaxy system at elevated substrate temperatures above 400°C without post-growth annealing. High-resolution micro-photoluminescence spectroscopy exhibits sharp excitonic emissions with lifetimes ranging from 0.7 to 1.1 ns. The coherence properties of the emitted photons are investigated by measuring the first-order field correlation function.

## Background

Various quantum emitters have been used to demonstrate single-photon emission, with single atoms or ions being the prototypical examples [1,2]. Other systems capable of single-photon generation are single molecules and single nanocrystals [3-5]. Their main drawback is their time stability, which is restricted by photo-bleaching and blinking [6].

Self-assembled semiconductor quantum dots (QDs) are the most promising zero-dimensional material system for the production of triggered single photons with high repetition rate [5,7,8], indistinguishable photons [9], and entangled photon pairs [10]. These are essential for quantum information and in novel photonic devices such as lasers, solar cells, detectors, and light-emitting diodes due to their ability to control their optical properties and the growth process. The Stranski-Krastanov (SK) method is the most used growth method on lattice-mismatched substrates.

Droplet epitaxy (DE) growth method was proposed [11] as an effective way of fabricating QDs without a wetting layer (WL) for both lattice-matched and lattice-mismatched epitaxial systems. Due to the lack of a WL, the DE QDs might have improved carrier confinement with high optical quality. DE offers advantages over the conventional SK method and thus a unique route to the fabrication of unpredicted nanostructures. Typically, DE uses low temperature for QD growth to prevent material redistribution [12,13]. However, the low-temperature growth hinders the good optical quality of the grown QD structures. Post-growth annealing processes were used for the restoration of the crystalline quality [14,15]. Post-growth annealing at 680°C for 1 h [14] improved the photoluminescence (PL) intensity, indicating efficient passivation of defects. It should be noted that some trap defects formed during the low-temperature growth cannot be recovered even by rapid thermal annealing at very high temperature. This problem could be avoided by growing the QDs at high substrate temperatures [16].

In this work, we report on the observation of triggered single-photon emission from a single InGaAs QD grown by DE mode on (100) GaAs substrates using a solid-source molecular beam epitaxy system at elevated substrate temperatures above 400°C. The morphological and structural properties are investigated. Their optical properties are characterized by single-dot spectroscopy and time-resolved spectroscopy. The excitonic states of a single QD are identified using power-dependent measurements on mesa-patterned samples.

## Methods

The investigated samples were grown on GaAs substrates by DE mode in a GEN II molecular beam epitaxy (MBE) system (Veeco Instruments Inc., Plainview, NY, USA) using an arsenic valved cracker cell for precise control of the As_4_ flux. The temperatures were measured using an optical pyrometer, and the QD formation was observed by reflection high-energy electron diffraction (RHEED). After removal of the native oxide at 600°C under As_4_ flux, a 200-nm GaAs buffer layer was grown at 590°C with a growth rate of 1 monolayer (ML)/s. Then, the InGaAs QDs have been grown under constant growth conditions. The substrate temperature was decreased to desired temperatures of 410°C and 500°C, and the arsenic valve was completely closed to reach a background pressure in the order of 1 × 10^−9^ Torr. A nominal amount of gallium equivalent to 1.75 MLs of GaAs was deposited on the GaAs buffer layer. Then, indium droplets equivalent to an amount of 4 MLs of InAs were deposited on the Ga-stabilized surface and supplied with an As_4_ molecular beam flux of 10^−5^-Torr beam equivalent pressure for a relatively short time to crystallize the indium droplets into InGaAs QDs. The time for QD formation was determined by RHEED. The QDs were capped with a 50-nm GaAs barrier layer with a growth rate of 1 ML/s without growth interruption. A second QD layer was grown on the top of the sample at the same growth conditions as the first QD layer for morphology investigations. Then, the samples were rapidly cooled down without arsenic pressure and taken out from the MBE chamber*.* For this study, we investigate two samples: Sample (A) and sample (B) consist of InGaAs QDs grown on a (100) GaAs substrate at 410°C and 500°C, respectively. It should be noted that no post-growth and rapid thermal annealing processes were applied in this study.

The PL measurements are performed at 5 K. The sample is mounted in a helium flow cryostat which can be moved using computer-controlled xy-linear stages with a spatial resolution of 100 nm. A microscope objective (NA = 0.7) is used to focus continuous wave (cw) laser with an excitation wavelength of 532 nm for micro-photoluminescence (μPL) measurements and a Ti:sapphire laser operating at 845 nm for photon correlation and time-resolved measurements. The same microscope objective was used to collect the QD emission. The collected luminescence was then spectrally filtered using a 0.75-m focal length monochromator equipped with a liquid nitrogen-cooled charge-coupled device for PL measurements. For photon statistics measurements, the PL light is directly sent to a modified Hanbury Brown and Twiss (HBT) correlation setup. The HBT consisted of a 50/50 non-polarizing beam splitter, two bandpass filters used to select a specific emission line, and two single-photon counting avalanche photodiodes (APDs), each providing a time resolution of approximately 700 ps. The lifetime measurements are performed using time-correlated single-photon counting technique. The signal is detected using a fast APD with a temporal resolution of about 50 ps.

## Results and discussion

From atomic force microscope studies (data not shown here), the QD density decreases from approximately 10^11^ to approximately 5 × 10^9^ cm^−2^ with increasing substrate temperature from 410°C to 500°C, respectively, due to the larger diffusion length of the indium atoms at higher substrate temperature which shows a behavior similar to SK growth mode. The optical properties of the QD ensemble characterized by macro-photoluminescence (not shown here) show that the emission wavelength of QDs formed at 410°C is blueshifted in comparison to that of QDs formed at 500°C due to the smaller size of dots. The energy peak and the full width at half maximum of a QD ensemble grown at 500°C are similar to those of QDs grown by SK which is an indication for the good optical quality of the QDs formed by DE. For the following, we focus on single-QD investigations.

**Figure 1:**
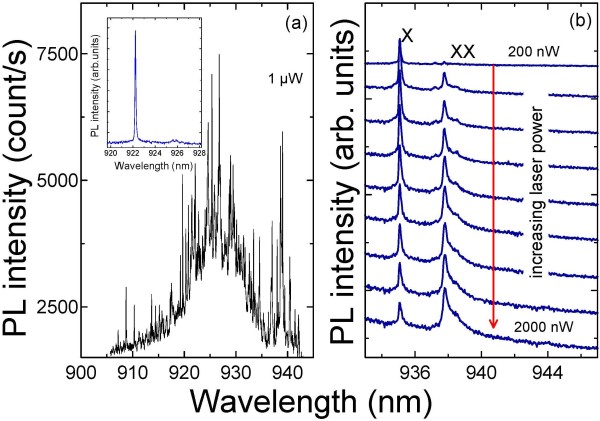
**Low-temperature photoluminescence taken from mesa structures. **(**a**) μPL spectrum of QDs taken from 10-μm mesa size grown by DE at 410°C; the inset is a typical PL spectrum of a single QD taken from 0.5-μm mesa size at very low laser excitation power. (**b**) PL spectra of a single QD under cw optical excitation, recorded for different laser excitation powers.

Figure 1a shows a PL spectrum of an ensemble of QDs taken from 10-μm mesa size grown by DE at 410°C. The spectrum exhibits many sharp emission peaks within 910 to 940 nm. To clearly distinguish the emission peaks from a single QD, we fabricated small mesa structures with size ranging from 200 to 700 nm and measured the μPL spectrum near 920 and 940 nm, where the density of QDs is relatively low. A typical PL spectrum of a single QD taken from 0.5-μm mesa size at very low laser excitation power is shown as inset of Figure 1a with a PL linewidth of around 110 μeV observed for single QDs, which is larger than those of the best SK-grown QDs. This could be ascribed to spectral diffusion related to the charging and discharging of trap defects in the QD surroundings. Figure 1b displays PL spectra of a single QD in 0.5-μm mesa size under cw optical excitation, recorded for different laser excitation powers. At low excitation power, only one excitonic line is seen which attributed to an exciton (X). As the power is increased, a second line appears assigned to a biexciton (XX) line. The intensity of the X line shows approximately linear power dependence, whereas the intensity of the XX line shows a superlinear increase (data not shown here). At high cw excitation powers, the X line reaches a saturation behavior before the XX line. It should also be noted that the biexciton binding energy is around 3-5 meV which is comparable to SK-grown QDs.

**Figure 2:**
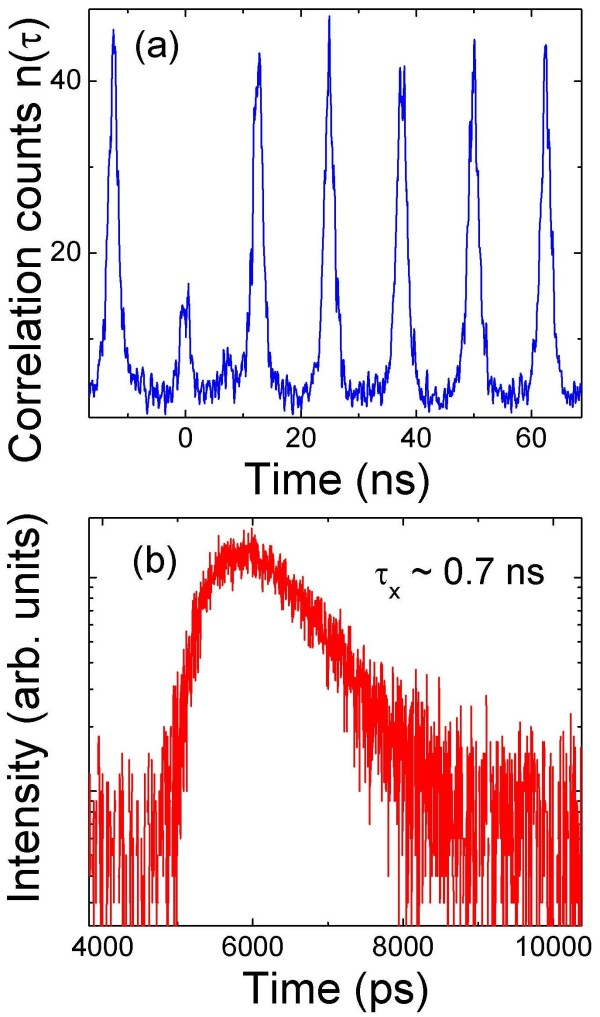
**Triggered single-photon and time-resolved PL measurement. **(**a**) Triggered single-photon emission of the X line from single QD grown by DE at 410°C; the corresponding PL spectrum is shown in Figure 1b taken at a relatively low laser excitation power of 500 nW. (**b**) The X lifetime.

The fact that at high excitation powers no groups of lines appear to higher energies in Figure 1b is strong evidence for the existence of only one pair of electron and hole levels in the QD. To demonstrate triggered single-photon generation, we measured the second-order correlation function *g*^(2)^(*τ*) of X photon under pulsed excitation. Figure 2a shows the measured unnormalized correlation function *n*(*τ*) of the X emission of the single QD. The corresponding PL spectrum is shown in Figure 1b. All displayed measurements were performed at relatively low excitation power. The measured *n*(*τ*) consists of series of correlation peaks separated by the repetition period. The central peak at *τ* = 0 ns of the QD X emission is suppressed with a value below 0.5, a signature of a single-photon emission. For perfect single-photon emission, the central peak is absent indicating the generation of only one photon per pulse. Time-resolved PL measurements are performed to understand the dynamics of the recombination process. Figure 2b shows the measured X lifetime of the single QD with a value of 0.7 ns, which is shorter compared to typical X lifetimes of single QDs grown by SK mode.

**Figure 3:**
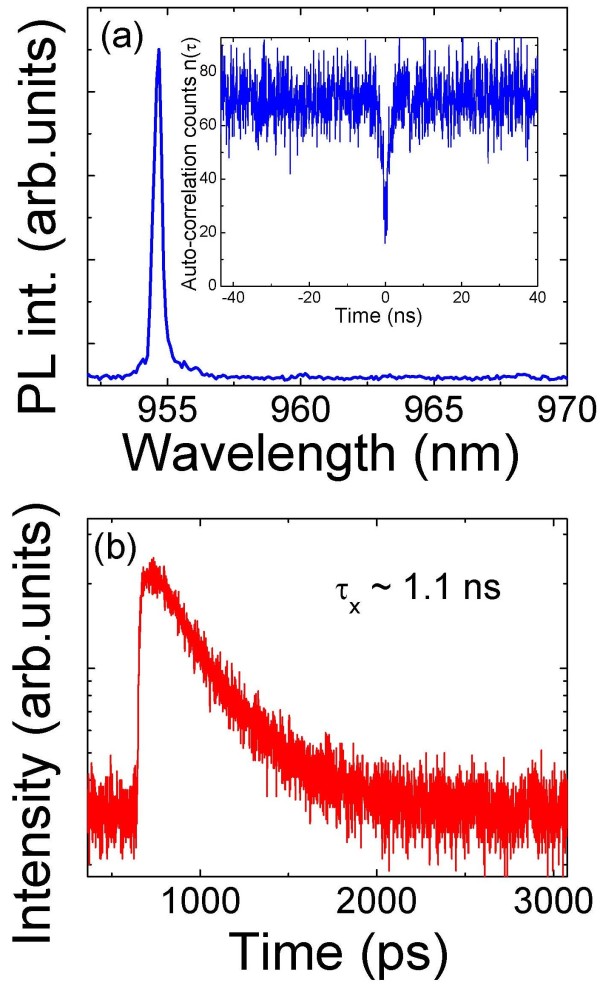
**Autocorrelation and time-resolved measurements. **(**a**) Autocorrelation measurement of the X line from a single QD grown by DE at 500°C. (**b**) The corresponding X lifetime at a laser excitation power of 500 nW.

Figure 3a displays a PL spectrum of single QDs grown at 500°C with an excitonic linewidth of about 80 μeV. This indicates good optical properties of the grown DE QDs but is larger than those of the best SK-grown QDs. As reported in previous works, the linewidth is attributed to spectral diffusion, caused by charge rearrangement in the QDs’ environment [17]. Further investigations are necessary to get better understanding of the mechanisms responsible for the line broadening. Autocorrelation measurements have also been performed to demonstrate single-photon generation under cw excitation of X line the single QD grown at 500°C. The inset of Figure 3a shows the measured unnormalized correlation function *g*^(2)^(*τ*) of the X QD emission at 5 K. The trace exhibits a clear dip in the correlation counts for the time delay *τ* = 0 ns, indicating a strong photon antibunching. The *g*^(2)^(0) < 0.5 is a signature of a single quantum emitter. The measured *g*^(2)^(0) does not reach its theoretical value of 0 because of the presence of uncorrelated background.

To confirm the improvement in optical quality, we have also performed time-resolved μPL measurements on single QDs in sample B. Figure 3b shows typical time-resolved PL of the X line of single QD. While the excitonic lifetimes of QDs in sample A are less than 0.8 ns, those of sample B are larger than 1.1 ns. These results confirm the improved optical quality of QDs grown at 500°C. This could be due to the removal of excess arsenic atoms which act as effective nonradiative recombination centers. The increased radiative lifetime for QDs grown at 500°C compared to QDs grown at 410°C could also be due to the increasing QD size, which has been predicted theoretically [18] and also reported experimentally [19]. This could likely be due to variation of the e-h overlap induced by different QD morphologies and by piezoelectric effects [20]. However, further investigations are necessary to classify the nature of the defects related to the excess arsenic incorporation by using low-density QD samples.

**Figure 4:**
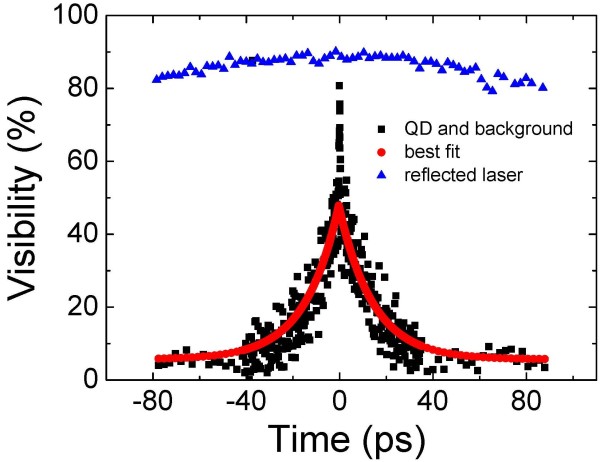
**Fourier transforms spectroscopy. **Visibility curves of the laser and the excitonic line from a single QD grown at 500°C.

We now report on the measurements of the first-order correlation function, *g*^(1)^(*τ*), which provides direct experimental access to the coherence properties of the emitter. In addition, time-resolved PL studies complement these measurements and allow the comparison of lifetime and coherence time. The PL emission from the QD is passed through a Michelson interferometer to carry out Fourier transform spectroscopy [21,22] and deduce the coherence time, *T*_2_, of the emitted photons. The visibility curves of the laser and the X line from the single QD grown at 500°C are plotted in Figure 4. The curves with points show the measurement data, and the solid line is an exponential fitting to the data. A *T*_2_ value of 35 ps is extracted from the fitting. The measured *T*_2_ value is much shorter compared to the radiative lifetime, *T*_1_, of approximately 1.1 ns, which could be due to dephasing that originated from fluctuating charges which accumulate in the vicinity of the dots. It should be noted that the samples used in this study were nano-patterned to have small-sized mesas ranging from 200 to 700 nm. Many charge-trapping defects can exist, especially at the sidewalls of the mesa structures, which generate stronger electric fields compared to bulk samples without mesa structures. As a result, these stronger fields appear as larger spectral diffusion. This partially explains the short *T*_2_ which could be due to dephasing. Further investigations to clarify this issue are necessary.

## Conclusions

In conclusion, we have demonstrated single-photon emission from single InGaAs QDs grown by droplet epitaxy at elevated substrate temperatures. Excitation power-dependent and time-resolved micro-photoluminescence (TRμPL) measurements were performed at low temperature to characterize the optical properties of the excitonic states. TRμPL measurements showed an increase in lifetime from 0.70 to 1.16 ns with the increasing substrate temperature, which could be related to the removal of excess arsenic atoms that act as nonradiative recombination centers and the increased QD sizes. The lifetime characteristics were attributed to good quantum confinement of carriers in DE-grown QDs.

## Competing interests

The authors declare that they have no competing interests.

## Authors’ contributions

VZ grew the samples with the guidance of JPR. TK, AWS, and TA performed the autocorrelation and lifetime measurements in the group of OB. MB conceived the experiment, carried out the micro-photoluminescence measurements, coordinated the study, and wrote the manuscript with the input from other authors. All authors read and approved the final manuscript.
